# Comparison of the Effects of Proximal Humeral Internal Locking System (PHILOS) Alone and PHILOS Combined with Fibular Allograft in the Treatment of Neer Three‐ or Four‐part Proximal Humerus Fractures in the Elderly

**DOI:** 10.1111/os.12564

**Published:** 2019-11-24

**Authors:** Lei Zhao, Yi‐min Qi, Lei Yang, Gang‐rui Wang, Sheng‐nai Zheng, Qiang Wang, Bin Liang, Chun‐zhi Jiang

**Affiliations:** ^1^ Department of Orthopaedic Surgery, Nanjing First Hospital Nanjing Medical University Nanjing China

**Keywords:** Anatomical allograft, Elderly, Fibular shaft, Proximal humeral internal locking system, Proximal humerus fractures

## Abstract

**Objective:**

To compare and analyze the clinical outcomes of the proximal humeral internal locking system (PHILOS) alone and the PHILOS combined with fibular allograft in the treatment of Neer three‐ and four‐part proximal humerus fractures (PHF) in the elderly.

**Methods:**

From January 2014 to January 2018, a total of 42 elderly patients with Neer three‐ or four‐part PHF admitted to our hospital were randomly divided into observation group and control group, with 21 patients in each group. The observation group was treated with the PHILOS combined with fibular allograft. The control group was treated with the PHILOS alone. Perioperative parameters and fracture classification were recorded in the two groups. Function results were assessed by Visual Analog Scale (VAS), Constant‐Murley score (CMS), American Shoulder and Elbow Surgeons (ASES) score, and the Disability of Arm‐Shoulder‐Hand (DASH) score. Radiological results were evaluated using the neck‐shaft angle (NSA) and humeral head height (HHH), and complications were also recorded in each group.

**Results:**

There were no significant differences between the two groups in terms of preoperative status, age, gender, cause of trauma, fracture site, and fracture classification. The average follow‐up time was 12 months. At the last follow‐up, the VAS and DASH observation groups were lower than the control group, and there was significant difference between the two groups (*P* < 0.05). The CMS and ASES were higher in the observation group than the control group, and there was significant difference between the two groups (*P* < 0.05). The mean difference in the NSA and HHH were lower in the observation group than the control group, and there was a significant difference between the two groups (*P* < 0.05). There was one postoperative complication in the observation group, which was humeral head avascular necrosis (AVN). There were seven postoperative complications in the control group, including three cases of humeral head collapse and three cases of screw cutout and one case of humeral head AVN. The incidence of postoperative complications in the observation group was significantly lower than the control group (*P* < 0.05), there was a significant difference between the two groups.

**Conclusions:**

For Neer three‐ or four‐part PHF in the elderly patients, PHILOS fixation with fibular allograft shows satisfactory short‐term results with respect to humeral head support and maintenance of reduction, and may reduce the incidence of complications associated with fixation using a PHILOS alone.

## Introduction

Proximal humeral fractures (PHF) account for 4%–5% of the whole body bone fractures^1^, and the incidence of PHF is increasing due to a high number of elderly osteoporotic patients^2^, making PHF the third most common injury among older people^3^. It has been reported that more than 80% of patients with minimally displaced PHF can be managed by nonoperation^4^, but approximately 20% of patients with displaced and comminuted fractures require surgery^5^. These fractures are difficult to treat, as it is unpredictable whether they will achieve stable fixation that maintains intra‐operative reduction. Operative management of PHF still remains challenging for orthopedic surgeons in the world^6^.

Although various surgical techniques have been described for the unstable PHF, proximal humeral internal locking systems (PHILOS) are increasingly popular for treating these fractures because they offer improved biomechanical properties by providing divergent and convergent fixed‐angle screws that improve fixation and pullout strength in osteoporotic bone^7^. It is also known to be clinically and biomechanically effective in elderly patients with PHF^8^. However, it is difficult to obtain stable fixation in osteoporotic patients even with PHILOS^9^. Some authors have also reported some complications, such as avascular necrosis (AVN), screw cutout, implant failure, plate impingement, head collapse, and infection^10^, ^11^. Good outcomes have been reported following the use of an associated intramedullary allograft^9^, ^12^. However, most previous studies are case series, and no comparative study has evaluated the clinical and radiological outcomes of PHILOS with and without an associated fibular allograft in elderly patients.

The aim of this study was to compare and analyze the clinical outcomes of the PHILOS alone and the PHILOS combined with fibular allograft in the treatment of Neer three‐ or four‐part PHF in the elderly. The hypothesis was that patients treated using the PHILOS combined with fibular allograft would have better clinical and radiological outcomes with lower complication rates than those treated using only the PHILOS alone.

### *Patients and Methods*

This research was a retrospective study and was approved by the Ethics Committee of Nanjing First Hospital, Nanjing Medical University. Written informed consents were obtained from all enrolled patients.

From January 2014 to January 2018, a total of 42 elderly patients with Neer three‐ or four‐part PHF admitted to Nanjing First Hospital were randomly divided into observation group and control group, with 21 patients in each group. The observation group was treated with the PHILOS combined with fibular allograft. The control group was treated with the PHILOS alone. Perioperative parameters and fracture classification were recorded in the two groups. The demographic characteristics of the patients in the two groups are shown in Table 1.

**Table 1:** Comparison of general data between the two groups of patients

Variables		Observation group(n = 21)	Control group(n = 21)	*χ*2/*t*‐value	*P*‐value
Gender	Male	11	12	0.096	0.757
	Female	12	9		
Mean age		68.8 ± 6.3	69.0 ± 7.2	0.114	0.901
Preoperative time (days)		4.2 ± 1.1	4.3 ± 1.2	0.281	0.780
Mechanism of injury	F	15	17	0.525	0.469
	TA	6	4		
Fracture site	Left	9	11	0.382	0.537
	Right	12	10		
Neer classification	Neer 3	14	15	0.111	0.739
	Neer 4	7	6		

Observation group: PHILOS combined with fibular allograft; Control group: PHILOS alone.

Inclusion criteria were as follows: (i) patients over 60 years of age; (ii) unilateral acute shift of Neer classification^13^ of three‐ or four‐part of the proximal humerus fracture; (iii) patient's fragments were either displaced more than 1.0 cm or angulated more than 45° and were preoperatively conformed by radiograph or computed tomography (CT) with three‐dimensional (3D) reconstructions; and (iv) there were no obvious surgical contraindications.

Exclusion criteria were as follows: (i) a history of shoulder surgery or chronic bone nonunion, there was severe soft tissue injury at the surgical site or systemic or local infection; (ii) pathological fracture or open fracture; (iii) complications of serious nervous or vascular injury; (iv) hypertensive patients with poor blood pressure control and diabetic patients with poor glycemic control; and (v) patients who refused to participate or failed to cooperate during the trial.

## Surgical Technique

A proximal humeral locking plate (PHILOS; DePuy Synthes, Pennsylvania, America) was used in all patients. A fibular allograft (Datsing, Beijing, China) was used in the observation group.

Step 1: All patients were placed in the beach chair position on a radiolucent operating table followed by cervical plexus anesthesia.

Step 2: A deltopectoral approach was performed for all patients. An approximate 15 cm skin incision was made. The insertion of the deltoid muscle was one‐half detached posteriorly subperiosteally.

**Figure 1:**
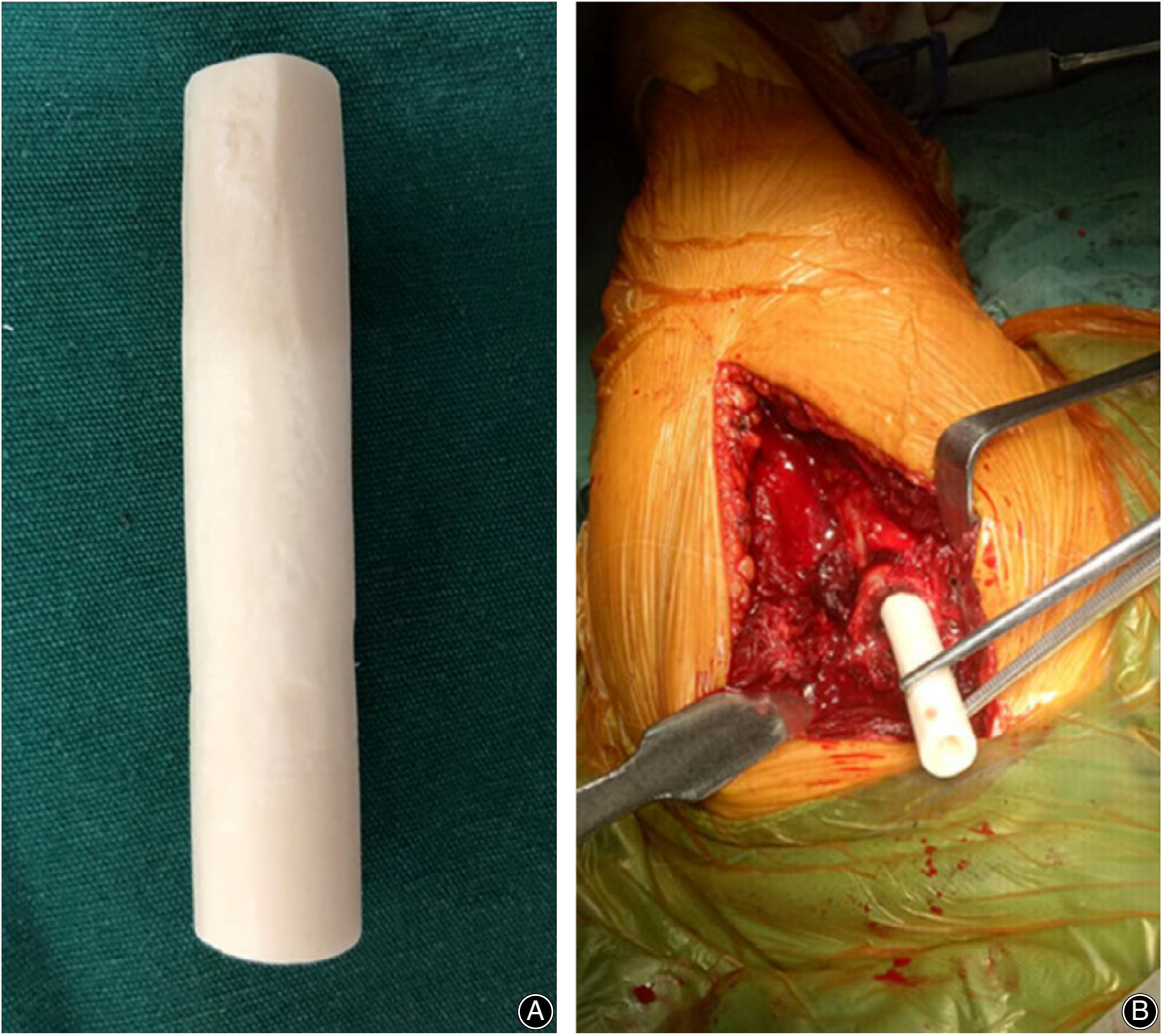
(A) Fibular allograft (12 mm×60 mm); (B) Insertion of fibular allograft.

**Figure 2:**
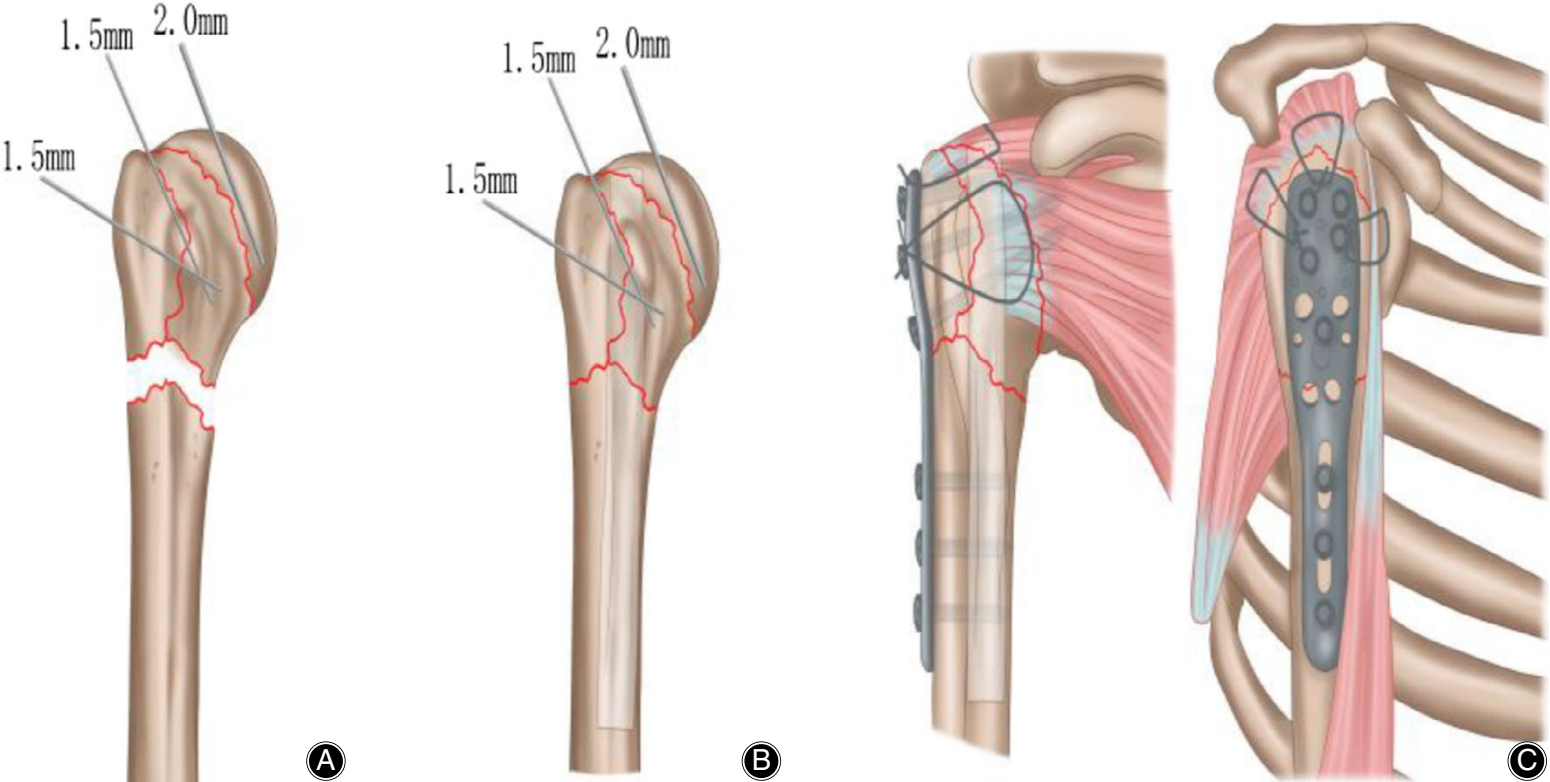
(A) (The illustration of the PHILOS combined with fibular allograft technology): A 2.0 mm Kirschner‐wire (K‐wire) was inserted into the humeral head to control rotation, and a periosteal elevator was used as a joystick at the fracture site to obtain reduction. After reduction of the greater tuberosity and humeral head, one or two 1.5 mm K‐wires were used for temporary fixation；(B) The allograft humerus is placed in the distal medullary cavity of the fracture, and the medial support is used to prevent the deformity and collapse of the humeral head in the long term; (C) Rotator cuff sutures were passed through the proximal humeral locking plate, and this was then slid from proximal to distal along the lateral aspect of the shaft, under the axillary nerve. The rotator cuff sutures were tied into place through eyelets on the plate, fixation was then obtained with screws and the wound was closed.

Step 3: Non‐absorbable sutures were passed through the junction of the greater tuberosity and the rotator cuff to promote mobilization and reduce fragmented tuberosities. A 2.0 mm Kirschner‐wire (K‐wire) was inserted into the humeral head to control rotation, and a periosteal elevator was used as a joystick at the fracture site to obtain reduction. After reduction of the greater tuberosity and humeral head, one or two 1.5 mm K‐wires were used for temporary fixation. The plate was placed in a position that did not impinge on the acromion. Indirect reduction was performed by inserting a cortical screw into the humeral shaft following the shape of the plate. In the observation group, fibular allograft (12 mm × 60 mm, Fig. 1) was inserted into the intramedullary canal distal to fracture site and was then driven back to the proximal humeral bone. Intramedullary fibular allograft was pushed onto the medial calcar to support the humeral head for prevention of varus displacement and deformity of the humeral head. The position varied depending on the configuration of the fracture. The graft was positioned vertically in valgus fractures and at a slightly oblique angle along the inferior cortex in the varus fractures. If anatomical reduction was achieved, locking screws were placed through the fibula into the humeral head and shaft to secure its position. Rotator cuff sutures were passed through the proximal humeral locking plate, and this was then slid from proximal to distal along the lateral aspect of the shaft, under the axillary nerve. The rotator cuff sutures were tied into place through eyelets on the plate, fixation was then obtained with screws and the wound was closed (Fig. 2).

### *Postoperative Rehabilitation*

The shoulders were immobilized in functional brace for between 4 and 6 weeks postoperatively. Patients received passive exercises immediately after the operation. About 4 weeks and 6 weeks later, active exercises including external and internal rotation were performed by/on all patients.

### *Clinical and Radiological Evaluation*

#### *Radiological Measurements*

**Figure 3:**
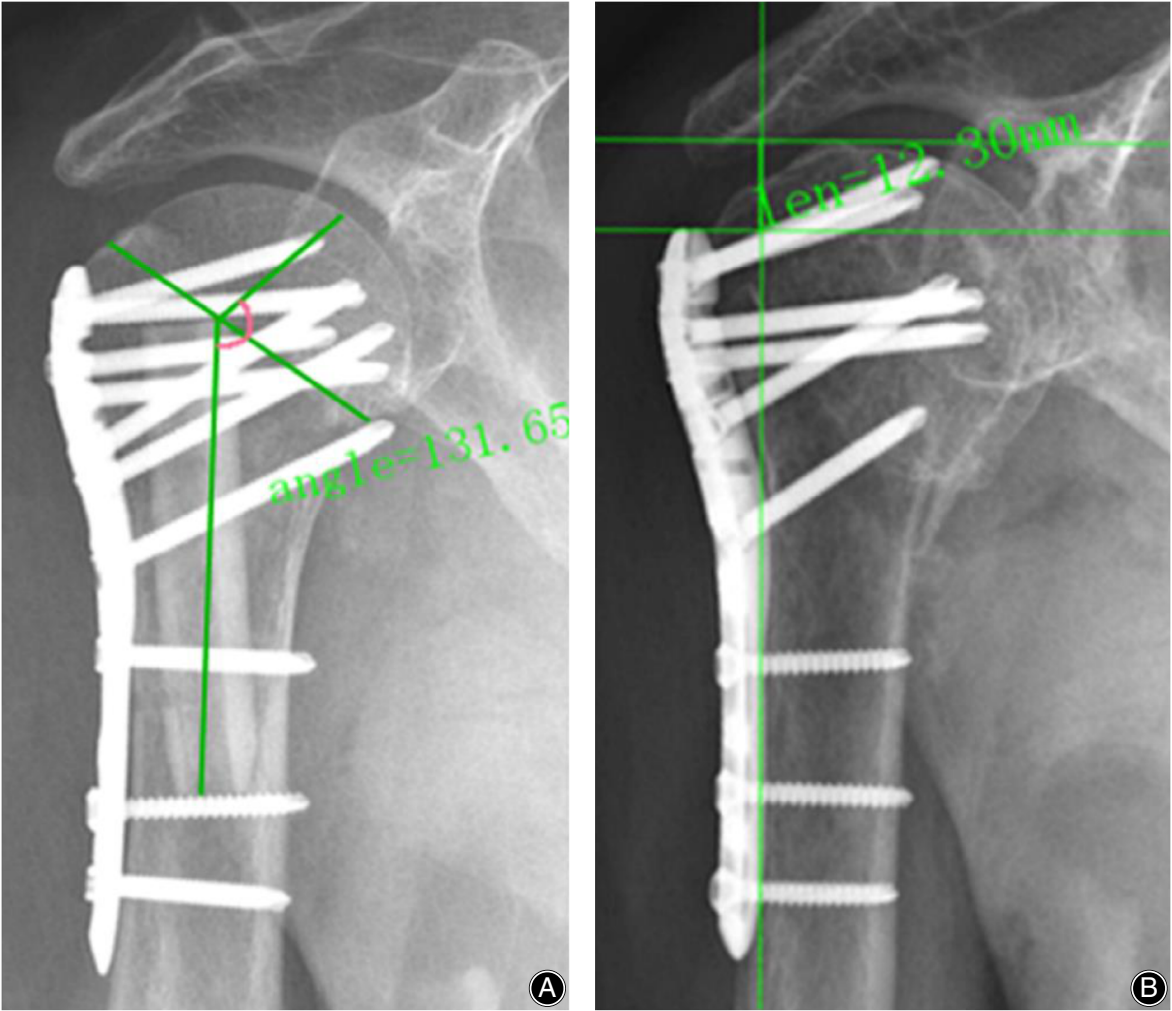
(A) The humeral neck‐shaft angle (NSA) was defined as the angle between a line that is perpendicular to the articular segment or atomic neck of the humerus and a line that bisects the humeral shaft; (B) The humeral head height (HHH) was defined as the distance between uppermost edge of the plate and the uppermost part of the humeral head.

Radiological evaluation was performed immediately after the operation and at the final follow‐up by measuring the neck‐shaft angle (NSA) and humeral head height (HHH) on anteroposterior views (Fig. 3).

The humeral neck‐shaft angle (NSA) was defined as the angle between a line that is perpendicular to the articular segment or atomic neck of the humerus and a line that bisects the humeral shaft by the measurement tools of the hospital's PACS system. The change in NSA was measured to assess whether the humerus had varus deformity.

HHH was defined as the distance between the upper end of the plate and the upper end of the humeral head by the measurement tools of the hospital's PACS system. The change in HHH was measured to assess the collapse of the humeral head. Comparing the immediate postoperative radiological findings with those at the final follow‐up, loss of fixation was defined if the varus NSA change was >5° or if HHH change was >3 mm^14^, ^15^.

#### *Postoperative Patient Follow‐Up*

Clinical evaluation was assessed at the final follow‐up using a Visual Analog Scale (VAS)^16^ score for pain, Constant‐Murley score (CMS)^17^, American Shoulder and Elbow Surgeons (ASES) score^18^, and Disability of Arm‐Shoulder‐Hand (DASH)^19^ score.

The VAS was used to assess the pain of the shoulder joint of the subject. The VAS total score is 10 points, with zero points indicating no pain and 10 points indicating unbearable pain.

The CMS score was 100 points, which consisted of pain (15 points), muscle strength (25 points), functional activity (20 points), and shoulder mobility (40 points). Higher scores indicate better functionality. Among them, objective evaluation indicators included shoulder mobility and muscle strength (65 points), and subjective evaluation indicators included pain and functional activities (35 points).

The rating scale of the ASES, which includes pain (50%) and life function (50%), is 100 points. The higher the score, the better the shoulder function.

The DASH scoring system consists of 30 items. Each of the five options corresponds to one to five points. When there are more than three missing items, the score is not calculated. The better the activity, the lower the score.

In addition, complications, such as AVN, screw cutout, implant failure, plate impingement, head collapse, and infection, were also recorded.

### *Statistical Analysis*

Statistical analysis was performed using SPSS v. 19.0 (SPSS Inc., Chicago, Illinois, US). Continuous variables, presented as the mean and standard deviation (SD), were compared by the Student *t* test to detect the group differences. Qualitative data of groups was compared by the *χ*2 test. *P* value <0.05 was considered as significant difference.

## Results

### *Intraoperative Radiological Outcomes*

The radiological outcomes are shown in Table 2. The average NSA immediately postoperatively in the observation group was 138.9° (118.9°–156.6°), the average NSA at the last follow‐up was 135.8° (118.4°–155.5°), and the mean difference in the NSA was 3.42 ± 0.71°. The average NSA immediately postoperatively in the control group was 135.4° (109.0°–155.8°), the average NSA at the last follow‐up was 125.8° (97.0°–149.7°), and the mean difference in the NSA was 9.82 ± 1.02°. The mean difference in the NSA between the values immediately postoperatively and those at the final follow‐up were significantly greater in the control group (*P* = 0.000).

**Table 2:** Radiological outcomes of the two groups

Outcomes	Observation group (n = 21)	Control group (n = 21)	*t*‐value	*P*‐value
Mean difference of NSA (°)	3.42 ± 0.71	9.82 ± 1.02	23.599	<0.001
Mean difference of HHH (mm)	2.14 ± 0.33	4.54 ± 0.42	2.429	<0.001

Observation group: PHILOS combined with fibular allograft; Control group: PHILOS alone; NSA, neck‐shaft angle; HHH, humeral head height.

The average HHH immediately postoperatively in the observation group was 12.3 mm (6.7 mm–15.7 mm), the average HHH at the last follow‐up was 10.2 mm (6.5 mm–13.5 mm), and the mean difference in the HHH was 2.14 ± 0.33 mm. The average HHH immediately postoperatively in the observation group was 12.2 mm (7.0 mm–18.5 mm), the average HHH at the last follow‐up was 9.0 mm (1 mm–15.1 mm), and the mean difference in the HHH was 4.54 ± 0.42 mm. The mean difference in the HHH between the values immediately postoperatively and those at the final follow‐up were significantly greater in the control group (*P* = 0.000).

### *Follow‐up Results*

The clinical outcomes are shown in Table 3. No patients were lost to follow‐up. The average follow‐up time was 12 months. All fractures healed clinically and radiologically. The VAS (*P* = 0.734) score was not significantly different between the two groups. The CMS (*P* = 0.020) score, ASES (*P* = 0.024) score, and DASH (*P* = 0.023) score were significantly different between the two groups at the final follow‐up.

**Table 3:** Clinical outcomes in the two groups

Outcomes	Observation group (n = 21)	Control group (n = 21)	*χ*2/*t*‐value	*P*‐value
VAS	1.14 ± 0.96	1.25 ± 1.12	0.342	0.734
CMS	86.00 ± 7.56	79.71 ± 9.14	2.429	0.020
ASES	87.76 ± 7.15	81.62 ± 9.62	2.349	0.024
DASH	17.95 ± 7.47	28.14 ± 8.27	4.190	<0.001
Complications	1	7	3.860	0.018

Observation group: PHILOS combined with fibular allograft; Control group: PHILOS alone; VAS, Visual Analog Scale; CMS, Constant‐Murley score; ASES, American Shoulder and Elbow Surgeons; DASH, Disability of Arm‐Shoulder‐Hand score.

### *Complications*

**Figure 4:**
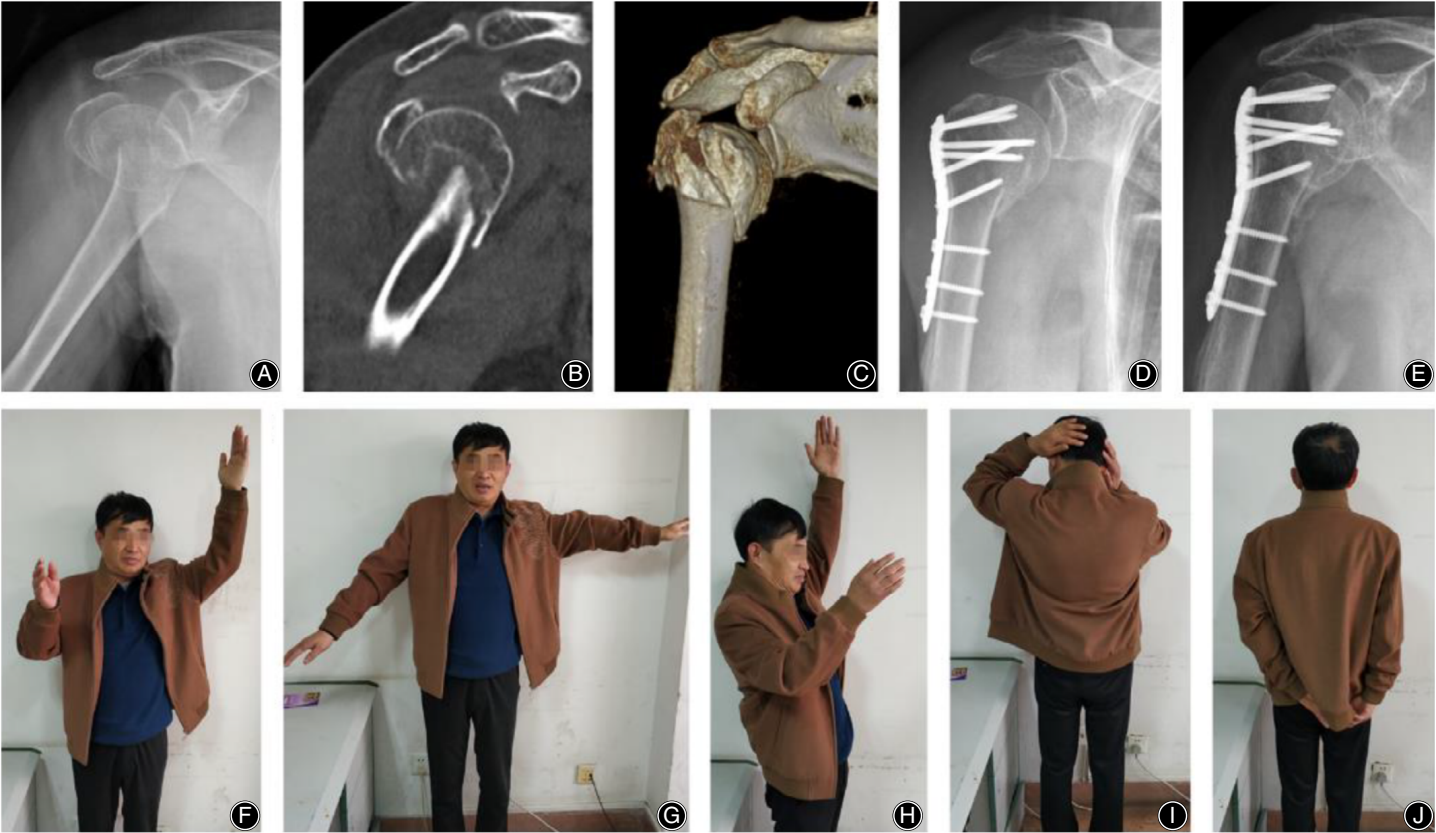
PHILOS fixation alone in a 65‐year‐old male patient with a four‐part proximal humeral fracture on the right side. (A) X‐ray film before surgery; (B) CT before surgery; (C) 3D CT before surgery; (D) X‐ray film after surgery; (E) X‐ray film 3 months after surgery; (F‐J) Function of patient's upper arm at the last follow‐up.

**Figure 5:**
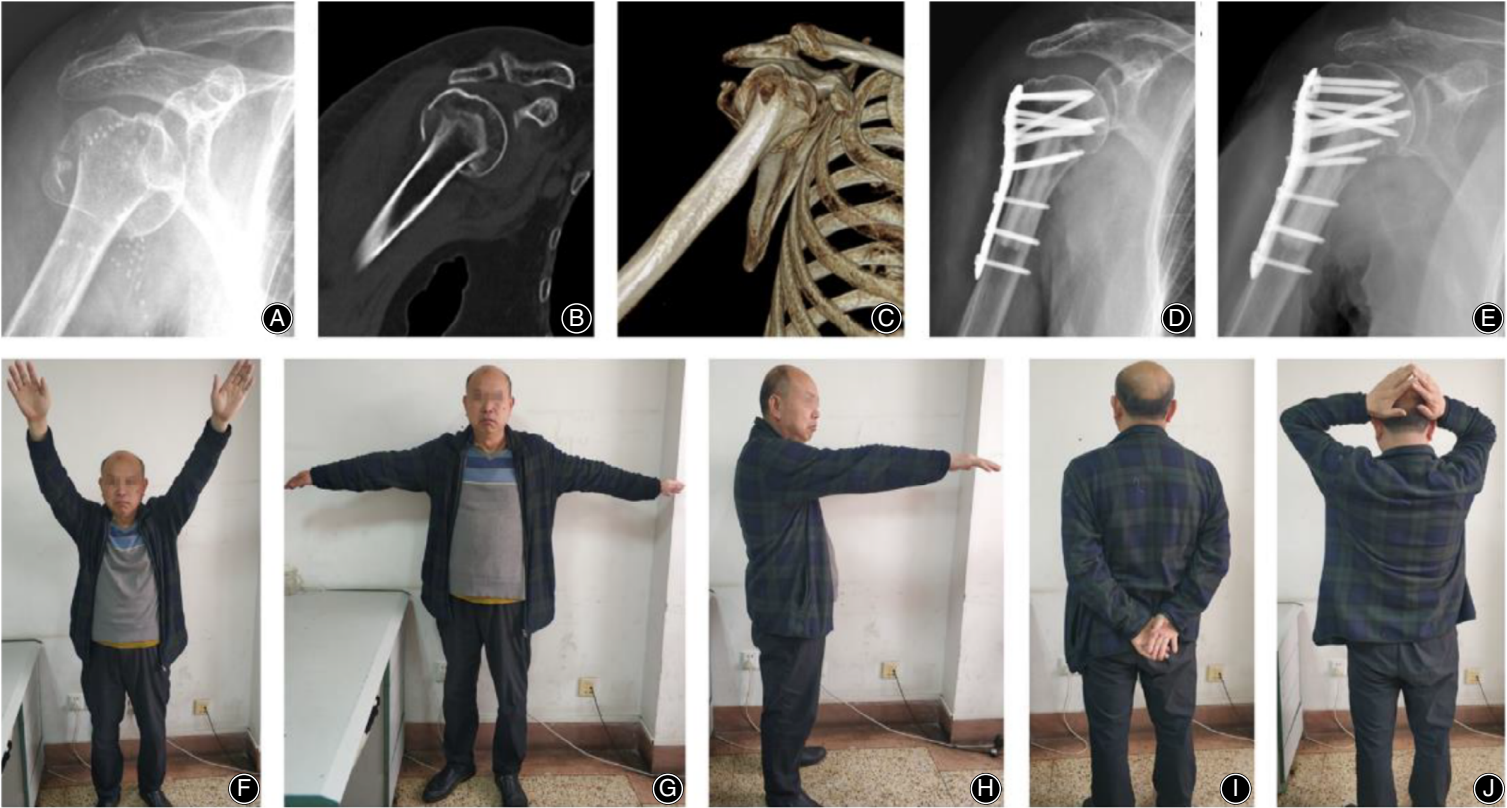
PHILOS fixation with fibular allograft in a 76‐year‐old male patient with a four‐part proximal humeral fracture on the right side. (A) X‐ray film before surgery; (B) CT before surgery; (C) 3D CT before surgery; (D) X‐ray film after surgery; (E) X‐ray film 3 months after surgery; (F–J) Function of patient's upper arm at the last follow‐up.

**Figure 6:**
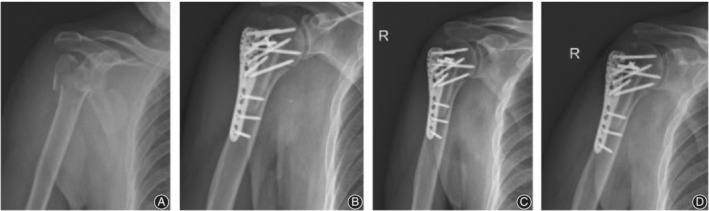
PHILOS fixation with fibular allograft in three patients with a four‐part proximal humeral fracture on the right side. (A) X‐ray film before surgery; (B) X‐ray film after surgery; (C) X‐ray film 1 month after surgery; (D) X‐ray film 3 months after surgery.

**Figure 7:**
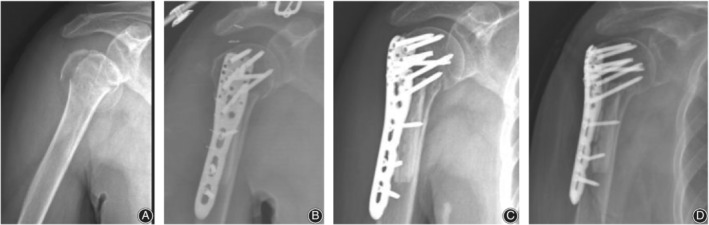
PHILOS fixation with fibular allograft in three patients with a four‐part proximal humeral fracture on the right side. (A) X‐ray film before surgery; (B) X‐ray film after surgery; (C) X‐ray film 1 month after surgery; (D) X‐ray film 3 months after surgery.

**Figure 8:**
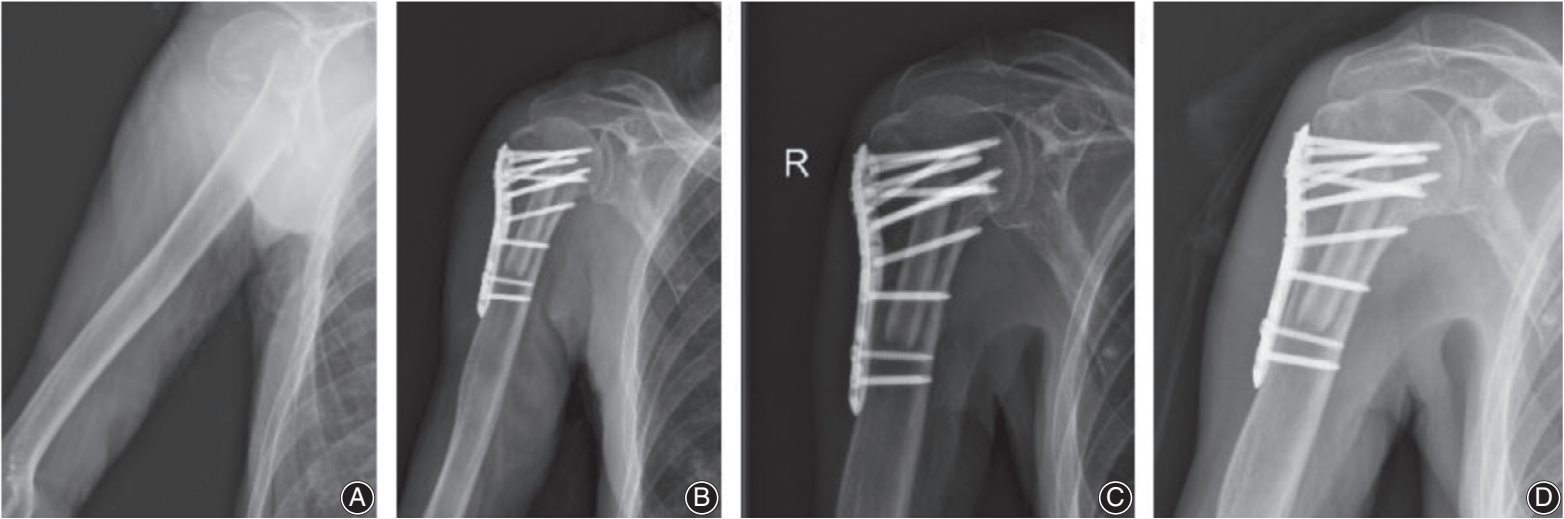
PHILOS fixation with fibular allograft in three patients with a four‐part proximal humeral fracture on the right side. (A) X‐ray film before surgery; (B) X‐ray film after surgery; (C) X‐ray film 1 month after surgery; (D) X‐ray film 3 months after surgery.

There were seven postoperative complications in the control group (Fig. 4), including three cases of humeral head collapse, three cases of screw cutout, and one case of humeral head AVN. There was one postoperative complication in the observation group (Fig. 5, 6, 7, 8), which was AVN.

## Discussion

PHF has been ranked as the third most frequent fracture and has a strong correlation with osteoporosis^20^. The management of these fractures depends on the vascular status, bone quality, fracture pattern, degree of commination, and patient factors. Non‐operative management is preferred for elderly patients and those with major comorbidities and for undisplaced fractures^21^. However, treating these fractures using non‐operative method requires a high level of patient compliance and it is associated with complications, like stiff shoulder and Sudeck's osteodystrophy^22^.

Although there are many surgical options available, PHILOS have consistently demonstrated biomechanical superiority over other forms of fixation in osteoporotic bone^23^, ^24^. Proponents of PHILOS fixation often cite better fixation, early mobilization, head preservation, restoration of range of motion, and satisfactory function as some of the major advantages of PHILOS construct^25^. But multiple studies have reported on the implant‐related complications associated with PHILOS fixation, most commonly, intra‐articular screw penetration, postoperative fracture displacement, and AVN^26^, ^27^, ^28^. A meta‐analysis of 12 studies with a total of 514 proximal humerus fractures treated with PHILOS fixation showed an overall complication rate of 49% and a 13.8% reoperation rate. The most common indication for reoperation involved intra‐articular screw perforation. The most common complications were varus malunion (16%), osteonecrosis (10%), intra‐articular screw penetration (8%), subacromial impingement (6%), and infection (4%)^29^.

The importance of reducing and maintaining the medial calcar to provide biomechanical support for a laterally placed plate has been recognized^30^, ^31^, ^32^. Researchers concluded that medial support screws played a key role in PHILOS fixation of displaced PHF, but a biomechanical study showed the addition of medial support screws had no effect on the stiffness of the medial cortex in cases with varus malunion^33^. In addition, a clinical study demonstrated that the place of calcar screws might lead to a high risk of humeral head necrosis^34^. Some investigators tried to use bone cement to strengthen the stability of PHILOS in PHF, and the data showed that a good clinical result with a decreasing complication rate^35^, but cement‐related heat injuries may exist^36^. Autologous bone grafting might be an alternative method for overcoming varus collapse. However, autologous bone grafting harvested from the patients themselves has some complications, such as vascular or neurologic injuries, deep infections at the donor site, and deep hematoma formation^37^.

Use of an endosteal strut allograft can re‐establish medial support, even in the comminuted osteoporotic bone commonly found in these patients. We considered that the fibula might be the most suitable donor bone for reconstructing the medial support in these types of fractures. Its length, geometrical shape, and mechanical strength might also be appropriate for these fractures. The cortical bone of the fibula provides immediate structural continuity and stability at the fracture site. When used as an intramedullary bone graft, it also has some osteogenic potential in addition to acting like a strut across the fracture site. Allograft is cancellous or corticocancellous chips or tricortical graft used as osteoconductive filler for metaphyseal defects. The fibular strut allograft may also minimize postoperative osteonecrosis by increasing the biomechanical strength of the construct and resisting a loss of reduction. Maintenance of reduction may permit revascularization of head pieces rendered ischemic at the time of injury. An important finding of this study is that the rate of varus malalignment and loss of HHH was significantly less when plate fixation was accompanied by a strut allograft.

The similarly good clinical outcomes in our study might be related with some factors. Fibular allograft used as volumetric filling in the bone void formed after reduction of humeral neck‐shaft angle could push the humeral head resistance to the force from the scapular fossa along with the screws, preventing the screw penetrating into the articular surface. Besides, this medial strut could prevent the varus placement of the head to diminish humeral head varus collapse and reduce the incidence of malunion. Anatomical medial strut with allograft bone has more potential to prevent humeral head varus displacement compared with the isolated fibula allograft. Anatomical allograft should be modified into a specific shape to fill the bone void according to the intramedullary geometry of the proximal humerus through computer virtual design with Pro‐E software. This kind of structural allograft provides enough medial stability and allows the formation of osteogenic tissue across a fracture site along with the surface of the allograft followed by bone formation. In addition, faster fracture healing could minimize articular segment AVN or collapse. Anatomical allograft is a plane contacted with the humeral head, and the support position could be pushed to the inferior medial point. However, isolated fibula was just a point‐to‐point support of the humeral head, and the support point is just at the line of extension of the intramedullary canal direction.

This study has several limitations. Firstly, it is not a randomized trial because the two techniques were performed at different times. Secondly, the study was limited by its retrospective design, which could introduce selection bias and the potential for confounding. Thirdly, although a large number of patients were included, this study had relatively low power to detect differences in forward elevation between the two groups. Subtle differences between the groups may not be identified in a single‐center study. A large‐scale, multicenter study would be required to investigate these clinical outcomes further.

## *Conclusions*

In conclusion, the present results showed that that patients treated by PHILOS combined with fibular allograft had a better functional outcome and a lower complication rate comparted to patients treated by PHILOS alone. Suitable void filler in the proximal humerus for supporting the head fragment, medial cortical bone and greater tuberosity might play a key role in reducing the incidence of complications in elderly patients, especially with osteoporosis.

## References

[1] Vachtsevanos L , Hayden L , Desai AS , Dramis A . Management of proximal humerus fractures in adults. World J Orthop, 2014, 5: 685–693. 25405098 10.5312/wjo.v5.i5.685 PMC4133477

[2] Wang MQ , Youssef T , Smerdely P . Incidence and outcomes of humeral fractures in the older person. Osteoporos Int, 2018, 29: 1601–1608. 29619542 10.1007/s00198-018-4500-2

[3] Cai M , Tao K , Yang C , Li S . Internal fixation versus shoulder hemiarthroplasty for displaced 4‐part proximal humeral fractures in elderly patients. Orthopedics, 2012, 35: 748. 10.3928/01477447-20120822-19 22955399

[4] Chivot M , Lami D , Bizzozero P , Galland A , Argenson JN . Three‐ and four‐part displaced proximal humeral fractures in patients older than 70 years: reverse shoulder arthroplasty or nonsurgical treatment. J Shoulder Elbow Surg, 2019, 28: 252–259. 30348542 10.1016/j.jse.2018.07.019

[5] Lee SH , Han SS , Yoo BM , Kim JW . Outcomes of locking plate fixation with fibular allograft augmentation for proximal humeral fractures in osteoporotic patients. Bone Joint J., 2019, 101‐B: 260–265. 10.1302/0301-620X.101B3.BJJ-2018-0802.R1 30813788

[6] Rabi S , Evaniew N , Sprague SA , Bhandari M , Slobogean GP . Operative vs non‐operative management of displaced proximal humeral fractures in the elderly: a systematic review and meta‐analysis of randomized controlled trials. World J Orthop, 2015, 6: 838–846. 26601066 10.5312/wjo.v6.i10.838 PMC4644872

[7] Gönç U , Atabek M , Teker K , Tanrıöver A . Minimally invasive plate osteosynthesis with PHILOS plate for proximal humerus fractures. Acta Orthop Traumatol Turc, 2017, 51: 17–22. 27866913 10.1016/j.aott.2016.10.003 PMC6197619

[8] Poole WEC , Wilson DGG , Guthrie HC , *et al* 'Modern' distal femoral locking plates allow safe, early weight‐bearing with a high rate of union and low rate of failure: five‐year experience from a United Kingdom major trauma centre. Bone Joint J, 2017, 99‐B: 951–957. 10.1302/0301-620X.99B7.BJJ-2016-0585.R1 28663403

[9] Matassi F , Angeloni R , Carulli C , *et al* Locking plate and fibular allograft augmentation in unstable fractures of proximal humerus. Injury, 2012, 43: 1939–1942. 22921382 10.1016/j.injury.2012.08.004

[10] Ricchetti ET , Warrender WJ , Abboud JA . Use of locking plates in the treatment of proximal humerus fractures. J Shoulder Elbow Surg, 2010, 19 Suppl: 66–75. 20188270 10.1016/j.jse.2010.01.001

[11] Frima H , Michelitsch C , Beks RB , Houwert RM , Acklin YP , Sommer C . Long‐term follow‐up after MIPO Philos plating for proximal humerus fractures. Arch Orthop Trauma Surg, 2019, 139: 203–209. 30421113 10.1007/s00402-018-3063-1

[12] Hardeman F , Bollars P , Donnelly M , Bellemans J , Nijs S . Predictive factors for functional outcome and failure in angular stable osteosynthesis of the proximal humerus. Injury, 2012, 43: 153–158. 21570073 10.1016/j.injury.2011.04.003

[13] Neer CS 2nd . Four‐segment classification of proximal humeral fractures: purpose and reliable use. J Shoulder Elbow Surg, 2002, 11: 389–400. 12195260 10.1067/mse.2002.124346

[14] Neviaser AS , Hettrich CM , Beamer BS , Dines JS , Lorich DG . Endosteal strut augment reduces complications associated with proximal humeral locking plates. Clin Orthop Relat Res, 2011, 469: 3300–3306. 21691909 10.1007/s11999-011-1949-0 PMC3210288

[15] Acklin YP , Stoffel K , Sommer C . A prospective analysis of the functional and radiological outcomes of minimally invasive plating in proximal humerus fractures. Injury, 2013, 44: 456–460. 23043975 10.1016/j.injury.2012.09.010

[16] Katz J , Melzack R . Measurement of pain. Surg Clin North Am, 1999, 79: 231–252. 10352653 10.1016/s0039-6109(05)70381-9

[17] Richards RR , An KN , Bigliani LU , *et al* A standardized method for the assessment of shoulder function. J Shoulder Elbow Surg, 1994, 3: 347–352. 22958838 10.1016/S1058-2746(09)80019-0

[18] Dabija DI , Jain NB . Minimal clinically important difference of shoulder outcome measures and diagnoses: a systematic review. Am J Phys Med Rehabil, 2019, 98: 671–676. 31318747 10.1097/PHM.0000000000001169 PMC6649681

[19] Prodinger B , Hammond A , Tennant A , Prior Y , Tyson S . Revisiting the disabilities of the arm, shoulder and hand (DASH) and QuickDASH in rheumatoid arthritis. BMC Musculoskelet Disord, 2019, 20: 41. 30683082 10.1186/s12891-019-2414-6 PMC6347833

[20] Launonen AP , Fjalestad T , Laitinen MK , *et al* Nordic Innovative Trials to Evaluate osteoPorotic Fractures (NITEP) Collaboration: The Nordic DeltaCon Trial protocol‐non‐operative treatment versus reversed total shoulder arthroplasty in patients 65 years of age and older with a displaced proximal humerus fracture: a prospective, randomised controlled trial. BMJ Open, 2019, 9: e024916. 10.1136/bmjopen-2018-024916 PMC6352806 30700485

[21] Koljonen AR , Fang C , Lau TW , *et al* Minimally invasive plate osteosynthesis for proximal humeral fractures. J Orthop Surg (Hong Kong), 2015, 23: 160–163. 26321541 10.1177/230949901502300208

[22] VL N , N B . Complex proximal humeral fracture fixation with PHILOS plate using Minimal Invasive Percutaneous Plate Osteosynthesis (MIPPO) technique: a series of 30 Patients. Malays Orthop J, 2018, 12: 20–24. 30112124 10.5704/MOJ.1807.004 PMC6092542

[23] Hettrich CM , Neviaser A , Beamer BS , Paul O , Helfet DL , Lorich DG . Locked plating of the proximal humerus using an endosteal implant. J Orthop Trauma, 2012, 26: 212–215. 22337487 10.1097/BOT.0b013e318243909c

[24] Foruria AM , Carrascal MT , Revilla C , Munuera L , Sanchez‐Sotelo J . Proximal humerus fracture rotational stability after fixation using a locking plate or a fixed‐angle locked nail: the role of implant stiffness. Clin Biomech, 2010, 25: 307–311. 10.1016/j.clinbiomech.2010.01.009 20153916

[25] Olerud P , Ahrengart L , Ponzer S , Saving J , Tidermark J . Internal fixation versus nonoperative treatment of displaced 3‐part proximal humeral fractures in elderly patients: a randomized controlled trial. J Shoulder Elbow Surg, 2011, 20: 747–755. 21435907 10.1016/j.jse.2010.12.018

[26] Schliemann B , Siemoneit J , Theisen C , *et al* Complex fractures of the proximal humerus in the elderly—outcome and complications after locking plate fixation. Musculoskelet Surg, 2012, 96: S3–S11. 22287062 10.1007/s12306-012-0181-8

[27] Micic ID , Kim KC , Shin DJ , *et al* Analysis of early failure of the locking compression plate in osteoporotic proximal humerus fractures. J Orthop Sci, 2009, 14: 596–601. 19802672 10.1007/s00776-009-1382-3

[28] Clavert P , Adam P , Bevort A , Bonnomet F , Kempf JF . Pitfalls and complications with locking plate for proximal humerus fracture. J Shoulder Elbow Surg, 2010, 19: 489–494. 19995683 10.1016/j.jse.2009.09.005

[29] Sproul RC , Iyengar JJ , Devcic Z , Feeley BT . A systematic review of locking plate fixation of proximal humerus fractures. Injury, 2011, 42: 408–413. 21176833 10.1016/j.injury.2010.11.058

[30] Jung WB , Moon ES , Kim SK , Kovacevic D , Kim MS . Does medial support decrease major complications of unstable proximal humerus fractures treated with locking plate. BMC Musculoskelet Disord, 2013, 14: 102. 23517539 10.1186/1471-2474-14-102 PMC3615943

[31] Krappinger D , Bizzotto N , Riedmann S , Kammerlander C , Hengg C , Kralinger FS . Predicting failure after surgical fixation of proximal humerus fractures. Injury, 2011, 42: 1283–1288. 21310406 10.1016/j.injury.2011.01.017

[32] Lescheid J , Zdero R , Shah S , Kuzyk PRT , Schemitsch EH . The biomechanics of locked plating for repairing proximal humerus fractures with or without medial cortical support. J Trauma, 2010, 69: 1235–1242. 20118814 10.1097/TA.0b013e3181beed96

[33] Bai L , Fu Z , An S , Zhang P , Zhang D , Jiang B . Effect of calcar screw use in surgical neck fractures of the proximal humerus with unstable medial support: a biomechanical study. J Orthop Trauma, 2014, 28: 452–457. 24662994 10.1097/BOT.0000000000000057

[34] Osterhoff G , Ossendorf C , Wanner GA , Simmen HP , Werner CM . The calcar screw in angular stable plate fixation of proximal humeral fractures–a case study. J Orthop Surg Res, 2011, 6: 50. 21943090 10.1186/1749-799X-6-50 PMC3189144

[35] Egol KA , Sugi MT , Ong CC , Montero N , Davidovitch R , Zuckerman JD . Fracture site augmentation with calcium phosphate cement reduces screw penetration after open reduction‐internal fixation of proximal humeral fractures. J Shoulder Elbow Surg, 2012, 21: 741–748. 22192764 10.1016/j.jse.2011.09.017

[36] Schliemann B , Wähnert D , Theisen C , *et al* How to enhance the stability of locking plate fixation of proximal humerus fractures? An overview of current biomechanical and clinical data. Injury, 2015, 46: 1207–1214. 25978864 10.1016/j.injury.2015.04.020

[37] Chen H , Ji X , Zhang Q , Liang X , Tang P . Clinical outcomes of allograft with locking compression plates for elderly four‐part proximal humerus fractures. J Orthop Surg Res., 2015, 10: 114. 26195025 10.1186/s13018-015-0258-9 PMC4509847

